# Recent changes in breast cancer incidence and risk factor prevalence in San Francisco Bay area and California women: 1988 to 2004

**DOI:** 10.1186/bcr1768

**Published:** 2007-09-25

**Authors:** Theresa HM Keegan, Ellen T Chang, Esther M John, Pamela L Horn-Ross, Margaret R Wrensch, Sally L Glaser, Christina A Clarke

**Affiliations:** 1 Northern California Cancer Center, 2201 Walnut Ave., Suite 300, Fremont, CA 94538, USA; 2 Division of Epidemiology, Department of Health Research and Policy, HRP Redwood Building, Stanford University School of Medicine, Stanford, CA 94305-5405, USA; 3 Departments of Neurological Surgery and Epidemiology and Biostatistics, University of California at San Francisco, 44 Page Street, Suite 503, San Francisco, CA 94102, USA

## Abstract

**Introduction:**

Historically, the incidence rate of breast cancer among non-Hispanic white women living in the San Francisco Bay area (SFBA) of California has been among the highest in the world. Substantial declines in breast cancer incidence rates have been documented in the United States and elsewhere during recent years. In light of these reports, we examined recent changes in breast cancer incidence and risk factor prevalence among non-Hispanic white women in the SFBA and other regions of California.

**Methods:**

Annual age-adjusted breast cancer incidence and mortality rates (1988 to 2004) were obtained from the California Cancer Registry and analyzed using Joinpoint regression. Population-based risk factor prevalences were calculated using two data sources: control subjects from four case-control studies (1989 to 1999) and the 2001 and 2003 California Health Interview Surveys.

**Results:**

In the SFBA, incidence rates of invasive breast cancer increased 1.3% per year (95% confidence interval [CI], 0.7% to 2.0%) in 1988–1999 and decreased 3.6% per year (95% CI, 1.6% to 5.6%) in 1999–2004. In other regions of California, incidence rates of invasive breast cancer increased 0.8% per year (95% CI, 0.4% to 1.1%) in 1988–2001 and decreased 4.4% per year (95% CI, 1.4% to 7.3%) in 2001–2004. In both regions, recent (2000–2001 to 2003–2004) decreases in invasive breast cancer occurred only in women 40 years old or older and in women with all histologic subtypes and tumor sizes, hormone receptor-defined types, and all stages except distant disease. Mortality rates declined 2.2% per year (95% CI, 1.8% to 2.6%) from 1988 to 2004 in the SFBA and the rest of California. Use of estrogen-progestin hormone therapy decreased significantly from 2001 to 2003 in both regions. In 2003–2004, invasive breast cancer incidence remained higher (4.2%) in the SFBA than in the rest of California, consistent with the higher distributions of many established risk factors, including advanced education, nulliparity, late age at first birth, and alcohol consumption.

**Conclusion:**

Ongoing surveillance of breast cancer occurrence patterns in this high-risk population informs breast cancer etiology through comparison of trends with lower-risk populations and by highlighting the importance of examining how broad migration patterns influence the geographic distribution of risk factors.

## Introduction

A striking feature of breast cancer epidemiology is its geographic variation in occurrence, with differences in invasive breast cancer incidence as high as 10-fold internationally [1] and two-fold among counties within the US [2]. At the highest end of these spectrums are incidence rates for non-Hispanic white women living in the San Francisco Bay area (SFBA) of California. Recently reported rates for this population were higher than those for other populations worldwide [1]. Rates have been reported to be further elevated among women in the small SFBA county of Marin, and these findings have received substantial public and scientific attention [3-5].

Prior studies have suggested that most [6,7], if not all [5,8], geographic variation in US breast cancer incidence relates to differences in the prevalence in women of established risk factors for breast cancer, including older age, non-Hispanic white race/ethnicity, US birthplace, low- or nulli-parity, late age at first birth, moderate to high consumption of alcohol, late age at menopause, and use of hormone therapy (HT). Many of these risk factors correlate with higher levels of education, income, and other metrics of socioeconomic status, which census data confirm to be more concentrated among SFBA residents, particularly non-Hispanic white women [9]. On this basis, it has been hypothesized that the elevated incidence of breast cancer in the SFBA may be largely attributable to the high prevalence in women of known breast cancer risk factors as opposed to geographically or environmentally unique features of the SFBA. However, to date, there have been few efforts to systematically document and compare specific risk factor prevalences in non-Hispanic white women in the SFBA with those in other populations [5,8].

With the recent and widely publicized changes in breast cancer incidence rates in this region (declines of 10% to 11% between 2001 and 2004 [10]) and elsewhere [11-13], the aim of this study is to determine whether incidence and mortality trends are correlated with changes in the population-level prevalence of breast cancer risk factors. In particular, HT use dropped substantially (60% to 70%) among middle-aged SFBA women [10] and in other populations [11,12] after the 2002 announcement by the Women's Health Initiative (WHI) that estrogen-progestin therapy increased the risk of breast cancer and heart disease [14]. A deeper understanding of detailed breast cancer incidence and risk factor prevalence patterns, particularly for distinct tumor subtypes, in this population at the high end of the international incidence spectrum can further inform the basis of geographic incidence variation, especially for years after the WHI announcement, and may offer important opportunities to generate new hypotheses about the etiology and possible prevention of breast cancer. With these goals in mind, we present risk factor prevalence data and the most current breast cancer incidence rates for the SFBA and the rest of California.

## Materials and methods

### Breast cancer incidence data

Breast cancer incidence and mortality data for non-Hispanic white females were obtained from the California Cancer Registry, October 2006 submission, for the period 1 January 1988 to 31 December 2004 and for white females (Hispanics and non-Hispanics) from the Surveillance, Epidemiology, and End Results (SEER) program of the National Cancer Institute (NCI) for the period 1 January 1973 to 31 December 2003 – the most recent year for which cancer data were complete at the time of this analysis. Analyses were based on incident cases of breast cancer (International Classification of Diseases-Oncology, 3rd edition [15] [ICD-O-3] site codes 50.0 to 50.9) occurring in six counties of the SEER and California Cancer Registry San Francisco/Oakland and San Jose/Monterey catchment regions (Alameda, Contra Costa, Marin, San Francisco, San Mateo, and Santa Clara counties), the rest of California, and the other eight original SEER regions (Connecticut, Detroit, Hawaii, Iowa, New Mexico, Seattle, Utah, and Atlanta). The populations of the six SFBA counties and the rest of California were 5,806,325 and 28,065,323, respectively. ICD-O-3 histology codes were used to distinguish the ductal (8500) and 'with lobular component' (8520, 8522, and 8524) histologic subtypes from other subtypes of breast cancer. Stage of disease at diagnosis was categorized as localized, regional, distant, or unstaged/not available. Tumor size was categorized into less than 2 cm and greater than or equal to 2 cm, and tumor marker variables were used to define estrogen receptor (ER) and progesterone receptor status as positive (+), negative (-), or missing. As receptor status was not reportable to SEER until 1990, trends by receptor status are limited to the period 1992–2004 due to higher completeness of the data [16-20]. From 1992 to 2004, hormone receptor status was missing for 16.9% of tumors in the SFBA and 29.2% of tumors in the rest of California; the percentage of missing hormone receptor status data decreased from 1992 to 1998 and was fairly stable after that time. Although we did not impute receptor status (as one prior study did [12]), the percentage of missing hormone receptor data did not vary from the period 2000–2001 to 2003–2004.

**Figure 1:**
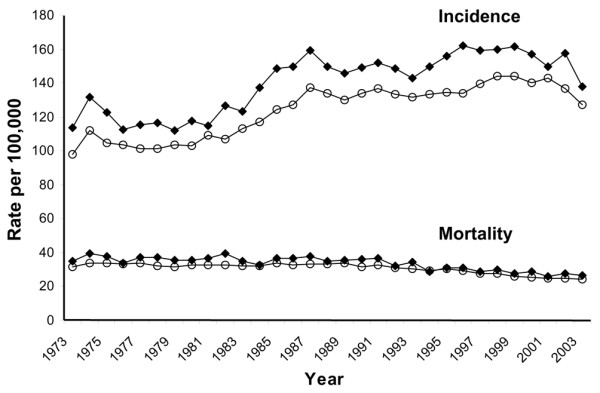
Trends in invasive breast cancer incidence and mortality rates among non-Hispanic and Hispanic white females in the San Francisco Bay area (◆) and other Surveillance, Epidemiology, and End Results (SEER) regions (o), 1973–2003.

The analyses presented in Figure 1 are based on all white women, both Hispanic and non-Hispanic, because ethnicity cannot be distinguished in the nine SEER regions for 1973–2003. All other analyses reported in this paper are restricted to non-Hispanic white women. Registry data on race/ethnicity are based on medical record information [21]. Population denominators were based on US Census Bureau estimates that were 'race-bridged' (that is, persons reporting two or more races in the 2000 census were allocated to a single race for comparability with prior census data) [22].

### Breast cancer risk factor prevalence estimates

Risk factor prevalence estimates for non-Hispanic white women between 35 and 74 years of age were derived from two population-based sources: (a) the California Health Interview Surveys (CHISs) conducted in 2001 and 2003 (source: 2001 and 2003 CHISs) and (b) controls identified through random digit dialing in four population-based case-control studies conducted between 1990 and 2000 in the SFBA (risk factor information was collected up to 1 year prior to selection dates, 1989 to 1999). We restricted analyses to the age group (35 to 74 years) common to the four population-based case-control studies: (a) Marin County Breast Cancer Study of Adolescent Risk Factors, which collected information up to 1 year prior to the selection dates, 1997 to 1999, from 297 controls who were residents of Marin County and who did not have a history of breast cancer [23]; (b) the San Francisco Bay Area Breast Cancer Study, which collected information up to 1 year prior to the selection dates, 1996 to 2000, from 564 controls who were residents of Alameda, Contra Costa, San Francisco, San Mateo, and Santa Clara counties and who did not have a history of breast cancer [24]; (c) Reproductive Factors in Hodgkin Lymphoma (HL) in Women, which collected information up to 1 year prior to the selection dates, 1990 to 1996, from 102 controls who were residents of Alameda, Contra Costa, Marin, Monterey, San Benito, San Francisco, San Mateo, Santa Clara, and Santa Cruz counties and who did not have a history of HL at interview [25]; women with a history of breast cancer at interview were excluded from the present analysis; and (d) the Bay Area Thyroid Cancer Study, which collected information up to 1 year prior to the selection dates, 1995 to 1998, from 205 controls who were residents of Alameda, Contra Costa, Santa Clara, San Francisco, and San Mateo counties and who did not have a history of thyroid cancer [26]; women with a history of breast cancer at interview were excluded from the present analysis. Response rates in the CHIS were 37.7% in 2001 [27] and 33.5% in 2003 [28] and were weighted to account for under-coverage and non-response biases [29]; response rates among controls in the population-based case-control studies ranged from 70% to 93% [23-26].

Breast cancer risk factors included in this study have been associated with breast cancer in prior studies [30] and either were available in the 2001 and/or 2003 CHIS or were assessed similarly in the four case-control studies. These factors included highest educational level attained, age at menarche (years), age at first live birth (years), number of live births, use of HT (ever, current, and duration in years), high alcohol consumption (two or more drinks per day in the past month), no vigorous or moderate physical activity in the past 30 days, and body mass index (BMI) (weight [kg]/height [m]^2^) stratified by age group (less than 50 years or greater than or equal to 50 years).

### Statistical analysis

#### Cancer incidence rates

SEER*Stat software [31] was used to compute average annual breast cancer rates age-adjusted to the 2000 US standard million population, associated standard errors and 95% confidence intervals, and annual percent changes (APCs). Age-adjusted rates were compared statistically using a Wald chi-square test of the difference between two rates [32], with *p *values of less than 0.05 considered significantly different. APCs across the period 1988 to 2004 were calculated by fitting a least squares regression line to the natural logarithm of the rates as the outcome variable, with calendar year as the predictor variable [33]. Time trends were analyzed using the NCI's Joinpoint software [34].

#### Risk factor prevalence

Prevalence estimates from the population-based controls were age-adjusted to the 2000 US standard million population using SAS version 9 software (SAS Institute Inc., Cary, NC, USA). Age-adjusted prevalence estimates from the 2001 and 2003 CHISs were obtained from the internet-based *Ask*CHIS application. CHIS data are weighted to the California Department of Finance estimates of the number of residents in each California county by age, race, and gender and the 2000 Census of Population counts from the US Census Bureau [35]. When the California and US census populations for non-Hispanic white women between 35 and 74 years of age were compared, the distributions were found to be similar (data not shown). The institutional review board of the Northern California Cancer Center approved this project.

## Results

**Figure 2:**
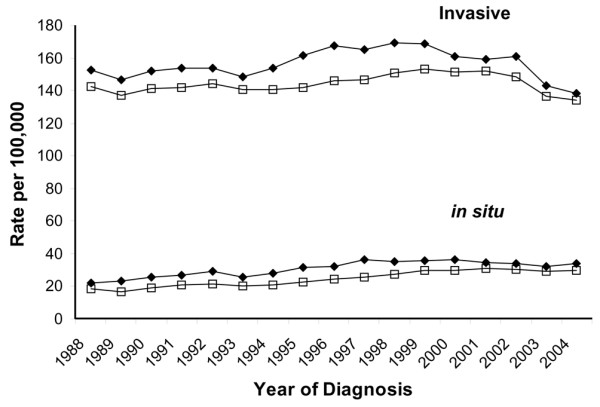
Trends in breast cancer *in situ *and invasive breast cancer incidence rates among non-Hispanic white females in the San Francisco Bay area (◆) and the rest of California (□), California Cancer Registry, 1988–2004.

**Figure 3:**
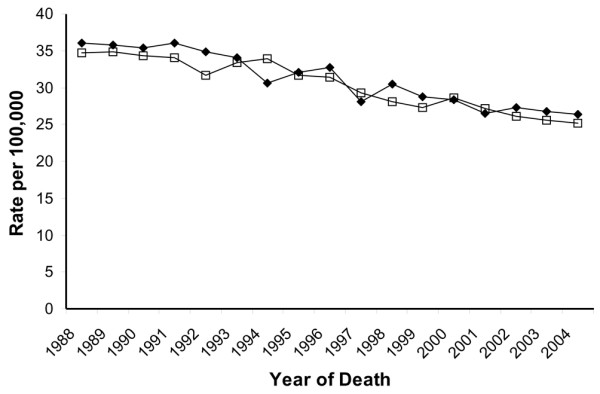
Trends in mortality rates among non-Hispanic white females in the San Francisco Bay area (◆) and the rest of California (□), California Cancer Registry, 1988–2004.

In white women (Hispanic and non-Hispanic), incidence rates of invasive breast cancer in the SFBA have been consistently higher than rates in other regions since the inception of continuous cancer surveillance by the SEER program in 1973 (Figure 1). The rates of both breast cancer *in situ *and invasive breast cancer were higher in non-Hispanic white women in the SFBA than in non-Hispanic white women in the rest of California (Figure 2). The APCs for breast cancer incidence and mortality rates are presented for each time period in Table 1. Invasive breast cancer incidence peaked in 1999 in the SFBA and in 2001 in the rest of California before declining (Figure 2; Table 1). Similar patterns in the incidence rate of invasive breast cancer occurred in most disease subgroups studied, including women 40 years old or older, all histologic subtypes, ER^+ ^tumors, localized and regional stage, and all tumor sizes (Table 1). On the other hand, the incidence rate of invasive breast cancer did not change in women under 40 years of age and, unlike overall incidence patterns, rates of ER^-^, distant and unstaged disease, and tumors of unknown size generally decreased over the study period in both regions. For breast cancer *in situ*, patterns differed, with incidence increases plateauing in 1998 in the SFBA but continuing to rise in the rest of California (Figure 2; Table 1). There were no major regional differences in breast cancer mortality trends (Figure 3; Table 1).

**Table 1:** Annual percent changes in breast cancer incidence and mortality rates in non-Hispanic white women, 1988–2004.

	San Francisco Bay area Annual percent change (95% CI)	The rest of California Annual percent change (95% CI)
Mortality rate	1988–2004: -2.20 (-2.57, -1.84)	1988–2004: -2.18 (-2.51, -1.84)
*In situ *incidence rate	1988–1998: +4.82 (3.82, 6.15) 1998–2004: -1.31 (-3.96, 1.41)	1988–2004: +3.94 (3.22, 4.66)
Invasive incidence rate	1988–1999: +1.32 (0.68, 1.97) 1999–2004: -3.61 (-5.61, -1.57)	1988–2001: +0.75 (0.42, 1.08) 2001–2004: -4.38 (-7.29, -1.38)
Histologic subtype		
Ductal	1988–1993: -1.59 (-3.04, -0.12) 1993–1997: +4.71 (1.29, 8.24) 1997–2004: -3.29 (-4.15, -2.43)	1988–1994: -0.58 (-1.72, 0.59) 1994–1999: +1.78 (-0.41, 4.02) 1999–2004: -3.51 (-4.98, -2.02)
With lobular component	1988–1999: +5.74 (4.54, 6.95) 1999–2004: -2.26 (-5.89, 1.51)	1988–2001: +5.75 (5.30, 6.19) 2001–2004: -5.30 (-9.03, -1.42)
Age at diagnosis (years)		
<40	1988–2004: +0.08 (-0.64, 0.79)	1988–2004: +0.28 (-0.25, 0.81)
40–69	1988–1999: +1.62 (0.81, 2.44) 1999–2004: -4.29 (-6.79, -1.71)	1988–2001: +0.91 (0.59, 1.23) 2001–2004: -4.14 (-7.00, -1.19)
≥70	1988–2002: +0.54 (0.04, 1.04) 2002–2004: -9.22 (-18.41, 1.02)	1988–2000: +0.68 (0.14, 1.22) 2000–2004: -4.21 (-6.93, -1.42)
ER status^a^		
ER^+^	1992–1996: +6.85 (3.00, 10.84) 1996–2002: +0.23 (-2.34, 2.87) 2002–2004: -10.36 (-20.18, 0.67)	1992–2001: +4.53 (3.71, 5.36) 2001–2004: -7.27 (-11.18, -3.20)
ER^-^	1992–2004: -2.11 (-2.86, -1.35)	1992–2004: -0.56 (-1.32, 0.21)
Missing	1992–2004: -4.15 (-5.74, -2.53)	1992–2004: -4.86 (-6.40, -3.29)
Stage at diagnosis		
Localized	1988–1998: +2.35 (1.60, 3.10) 1998–2004: -3.27 (-4.80, -1.72)	1988–2000: +1.36 (0.97, 1.75) 2000–2004: -3.71 (-5.67, -1.71)
Regional	1988–1993: -2.26 (-5.31, 0.89) 1993–2001: +1.96 (0.05, 3.91) 2001–2004: -7.13 (-13.48, -0.31)	1988–1996: -1.10 (-2.05, -0.14) 1996–2001: +4.06 (1.20, 7.01) 2001–2004: -6.34 (-10.38, -2.11)
Distant	1988–2004: -1.04 (-2.24, 0.17)	1988–2004: -1.17 (-1.65, -0.68)
Unstaged/Not available	1988–2004: -7.44 (-10.33, -4.46)	1988–2002: -4.24 (-5.52, -2.95) 2002–2004: -31.80 (-48.75, -9.25)
Tumor size		
<2 cm	1988–1999: +3.29 (2.60,3.99) 1999–2004: -3.70 (-5.82, -1.54)	1988–2001: +2.93 (2.46, 3.41) 2001–2004: -5.88 (-9.91, -1.66)
≥2 cm	1988–1993: -1.40 (-3.51, 0.75) 1993–1998: +3.10 (0.00, 6.30) 1998–2004: -4.12 (-5.67, -2.54)	1988–1992: -2.39 (-4.59, -0.15) 1992–2001: +0.38 (-0.41, 1.17) 2001–2004: -2.91 (-6.33, 0.65)
Unknown	1988–2004: -7.28 (-8.41, -6.13)	1988–2004: -6.45 (-8.34, -4.52)

^a^Annual percent change calculated for the period 1992–2004. CI, confidence interval; ER, estrogen receptor. Data from the California Cancer Registry.

From 2000–2001 to 2003–2004, rates of breast cancer *in situ *and invasive breast cancer incidence significantly decreased in the SFBA and the rest of California, as did mortality rates in the non-SFBA regions of California (Table 2). Decreases in invasive incidence rates were evident in most subgroups, except for women under 40 years of age and distant stage of disease at diagnosis in the rest of California. Between these two time periods, the percentage decrease in invasive breast cancer was slightly higher in SFBA women (12.0%) than women in the rest of California (10.9%), as were the decreases in ER^+ ^tumors (14.3%, SFBA; 12.9%, the rest of California), but not tumors less than 2 cm in diameter (11.6%, SFBA; 12.9%, the rest of California). In 2003–2004, incidence rates of breast cancer *in situ *and invasive breast cancer remained significantly higher in the SFBA than in the rest of California by 12.5% and 4.2%. (In 2000–2001, rates of breast cancer *in situ *and invasive breast cancer were 16.7% and 5.5% higher in the SFBA than in the rest of California.)

**Table 2:** Two-year average annual breast cancer incidence and mortality rates in non-Hispanic white women

	San Francisco Bay area		The rest of California	
	
	2000–2001 Rate (95% CI)	2003–2004 Rate (95% CI)	2000–2001 Rate (95% CI)	2003–2004 Rate (95% CI)
Mortality rate	27.4 (25.7–29.2)	26.5 (24.9–28.3)	27.8 (27.1–28.6)	25.4 (24.6–26.1)
*In situ *incidence rate	35.6 (33.6–37.7)	33.2 (31.3–35.2)	30.5 (29.7–31.4)	29.5 (28.7–30.4)
Invasive incidence rate	160.0 (155.9–164.3)	140.8 (136.9–144.8)	151.7 (149.9–153.6)	135.1 (133.4–136.9)
Histologic subtype^a^				
Ductal	110.9 (107.5–114.5)	97.0 (93.7–100.3)	98.9 (97.4–100.4)	88.4 (87.0–89.8)
With lobular component	29.6 (27.8–31.5)	26.9 (25.2–28.7)	30.4 (29.6–31.2)	27.0 (26.2–27.8)
Age at diagnosis (years)^a^				
<40	13.1 (11.5–15.0)	14.0 (12.1–16.1)	13.0 (12.2–13.9)	13.0 (12.2–13.9)
40–69	305.5 (295.4–315.8)	263.1 (253.9–272.5)	287.3 (282.8–291.9)	255.5 (251.3–259.7)
≥70	535.3 (511.7–559.8)	475.7 (452.4–499.9)	511.6 (501.3–522.0)	447.8 (438.0–457.7)
ER/PR status^a^				
ER^+^/PR^+^	96.0 (92.7–99.3)	83.7 (80.7–86.8)	76.0 (74.7–77.3)	65.9 (64.7–67.2)
ER^+^/PR^-^	19.5 (18.0–21.0)	15.3 (14.1–16.7)	15.9 (15.3–16.5)	14.1 (13.5–14.6)
ER^-^/PR^+^	2.3 (1.8–2.9)	1.0 (0.7–1.4)	1.9 (1.7–2.1)	1.3 (1.1–1.5)
ER^-^/PR^-^	21.6 (20.1–23.3)	20.3 (18.8–21.9)	20.5 (19.8–21.3)	19.6 (18.9–20.2)
Missing	20.7 (19.3–22.3)	20.3 (18.9–21.9)	37.4 (36.5–38.4)	34.3 (33.4–35.1)
Stage at diagnosis^a^				
Localized	103.4 (100.1–106.8)	93.1 (89.9–96.4)	95.0 (93.5–96.5)	84.6 (83.3–86.0)
Regional	48.4 (46.1–50.8)	40.5 (38.4–42.7)	47.8 (46.8–48.9)	42.0 (41.0–43.0)
Distant	6.0 (5.2–6.9)	5.3 (4.5–6.1)	5.4 (5.0–5.8)	5.5 (5.2–5.9)
Unstaged/Not available	2.3 (1.8–2.8)	1.5 (1.1–1.9)	3.4 (3.2–3.7)	2.2 (2.0–2.5)
Tumor size				
<2 cm	93.4 (90.2–96.7)	82.6 (79.6–85.7)	84.8 (83.4–86.2)	73.9 (72.6–75.2)
≥2 cm	58.1 (55.6–60.7)	51.6 (49.2–54.1)	58.2 (57.0–59.4)	53.5 (52.4–54.6)
Unknown	8.5 (7.6–9.5)	6.1 (5.3–6.9)	8.8 (8.3–9.2)	7.1 (6.7–7.5)

All rates per 100,000 and age-adjusted to the 2000 US age standard. ^a^Invasive breast cancer. CI, confidence interval; ER, estrogen receptor. Data from the California Cancer Registry.

Compared with non-Hispanic white women in the rest of California, non-Hispanic white women living in the SFBA were more likely to have graduated from college, to have a BMI below 25 kg/m^2 ^(women under 50 years of age), to have been physically active in the past 30 days, to have consumed alcohol in the past month (particularly two or more drinks per day), and to have had their first child after the age of 30 years or to be nulliparous (Table 3). From 2001 to 2003, CHIS data show that the percentage of women who graduated from college significantly increased in the SFBA and that the percentage of women with less than a high school education decreased in the rest of California. (There were no statistically significant changes in the percentages of women with higher levels of education in the rest of California.) Whereas there were no changes in the prevalence of women who drank alcohol in the past month, the prevalence of consumption of two or more alcoholic drinks per day increased among women in the rest of California, but not in the SFBA, between 2001 and 2003.

**Table 3:** Prevalence of breast cancer risk factors in non-Hispanic white women 35 to 74 years of age

Patient characteristics	Population-based control series (1989–1999)	California Health Interview Survey			
	
	SFBA (*n *= 1,168)	2001		2003	
		
		SFBA	California^a^	SFBA	California^a^
Education					
Some high school or less	3.2 (2.2–4.2)	2.3 (1.2–3.3)	7.5 (6.7–8.3)	2.9 (1.5–4.4)	6.2 (5.4–7.0)
High school graduate	20.7 (17.9–23.4)	18.1 (16.0–20.2)	23.3 (22.4–24.3)	15.4 (13.3–17.5)	23.8 (22.6–25.0)
Some college	27.7 (24.5–30.9)	27.0 (24.6–29.3)	34.4 (33.3–35.5)	25.1 (22.6–27.7)	34.3 (33.1–35.6)
College graduate or higher	46.7 (42.5–50.9)	52.7 (50.0–55.3)	34.8 (33.7–35.9)	56.5 (53.6–59.4)	35.6 (34.3–36.9)
Missing	1.6				
Body mass index <25 in women <50 years	64.4 (56.6–72.1)	64.1 (60.4–67.7)	58.0 (56.2–59.7)	63.9 (59.7–68.1)	57.2 (55.1–59.3)
Body mass index ≥ 30 in women ≥ 50 years	20.9 (17.5–24.2)	21.7 (18.5–24.8)	21.5 (20.2–22.9)	21.1 (17.7–24.5)	22.9 (21.4–24.4)
History of breast cancer in mother, sister, or daughter	13.9 (11.6–16.1)	^b^	^b^	^b^	^b^
Ever had a breast biopsy that was not cancer	11.7 (9.8–13.5)	^b^	^b^	^b^	^b^
Ever had radiation treatment to the chest	2.0 (1.2–2.7)	^b^	^b^	^b^	^b^
No vigorous/moderate physical activity in past 30 days	^b^	19.8 (17.7–22.0)	23.4 (22.4–24.4)	^b^	^b^
Drank alcohol in past month	^b^	71.3 (68.9–73.8)	61.3 (60.1–62.4)	69.8 (67.0–72.7)	61.0 (59.7–62.3)
2+ drinks per day^c^		27.6	23.6	28.0	25.9
Age at menarche <12 years	21.0 (18.2–23.8)	18.5 (16.4–20.6)	19.4 (18.5–20.4)	^b^	^b^
Age at first live birth ≥ 30 years	20.7 (17.8–23.7)	20.4 (18.3–22.5)	13.4 (12.6–14.2)	^b^	^b^
Number of live births					
0	25.0 (21.9–28.1)	25.3 (23.2–27.4)	17.3 (16.5–18.2)	^b^	^b^
1–2	48.2 (43.9–52.6)	51.4 (48.7–54.0)	48.0 (46.8–49.2)		
≥3	26.7 (23.8–29.7)	23.3 (21.0–25.6)	34.7 (33.6–35.8)		
Hormone therapy use					
Never	53.7 (48.8–58.5)	^b^	^b^	^b^	^b^
<5 years	21.2 (18.6–23.8)				
≥5 years	22.8 (20.3–25.3)				
Missing	2.3				
Ever took hormone supplements for menopause symptoms	^b^	^b^	^b^	29.8 (26.7–33.0)	34.5 (32.9–36.0)
Currently takes hormone supplements for menopause symptoms	^b^	34.9 (32.1–37.7)	37.0 (35.8–38.2)	16.7 (14.2–19.1)	21.6 (20.4–22.7)
Among women without a hysterectomy^d^		20.4	18.7	4.9	5.2
Mammogram screening history					
2 years or less	^b^	72.9 (70.5–75.2)	70.4 (69.3–71.5)	71.0 (68.3–73.7)	71.6 (70.3–72.8)
More than 2 years ago		12.1 (10.4–13.8)	13.7 (12.9–14.5)	12.9 (10.9–14.9)	13.4 (12.5–14.3)
Never had a mammogram		15.1 (13.1–17.0)	15.9 (15.0–16.8)	16.1 (13.8–18.3)	15.0 (14.0–16.1)

^a^Excluding the SFBA. ^b^Not available in the population-based control series or *Ask*CHIS. ^c^Calculated by dividing the number of women drinking 2+ drinks per day in the past 30 days by the total number of women (that is, the number of women who did and did not drink alcohol in the past 30 days). ^d^Calculated by dividing the number of women who did not have a hysterectomy and reported using hormone supplements by the total number of women (that is, the number of women who did and did not take hormone supplements for menopause symptoms). SFBA, San Francisco Bay area.

Based on 2001 CHIS data, significantly more women in the SFBA than in the rest of California had undergone mammographic screening within the last 2 years. However, based on 2003 data, there were no regional differences in mammography. To estimate combined estrogen and progestin HT use, we examined CHIS estimates of any HT use in non-pregnant women 40 years old or older who did not report a hysterectomy [36]. Compared with women in other regions of California, higher percentages of SFBA women used combined estrogen and progestin HT in 2001. In 2003, however, the percentages of women using combined estrogen and progestin HT were comparable in the two regions. From 2001 to 2003, CHIS data show that the percentage of women taking HT significantly decreased in both regions. According to control data from population-based case-control studies from 1989–1999 (which are not estimates of combined estrogen and progestin HT use), the prevalence of SFBA women who had ever used HT (44.0%) was higher than that among 2003 CHIS participants in either the SFBA (29.8%) or the rest of California (34.5%).

## Discussion

To identify new causes of breast cancer, epidemiological studies should fully characterize and, ideally, maximize the distribution of candidate risk factors in the population under study. With this goal in mind, we examined recent breast cancer incidence characteristics and trends over a 17-year period in one of the highest-risk populations in the breast cancer literature: non-Hispanic white women in the SFBA. We found that rates of invasive breast cancer increased from 1988 to approximately 1999 and decreased thereafter in women 40 years old or older. From 2000–2001 to 2003–2004, decreases in invasive breast cancer rates also occurred in women with all histologic subtypes, tumor sizes, hormone receptor-defined tumors, and localized and regional disease. A recent analysis in SEER also found decreases in tumors less than 2 cm, hormone receptor-defined tumors, and local and regional disease from 1999/2000 to 2003 [13]. Although similar trends were observed among non-Hispanic white women in the rest of California in our study, incidence and mortality rates of breast cancer were consistently higher in the SFBA than in the rest of California or in other SEER regions across all time periods. Even after the recent declines in 2003–2004, incidence rates of breast cancer *in situ *and invasive breast cancer were 12.5% and 4.2% higher, respectively, as compared with 16.7% and 5.5% higher in 2000–2001, among SFBA women than among women in the rest of California. Our age-specific incidence trends are similar to recent reports of declines in US [12,13,37,38] and German [11] women.

Contemporaneous population-based data on breast cancer risk factors from CHIS and case-control studies provide further evidence that in SFBA non-Hispanic white women, there is a higher prevalence of certain breast cancer risk factors [30], including advanced education, lower BMI among women younger than 50 years old, nulliparity, late age at first birth, use of estrogen plus progestin HT in 2001, and alcohol consumption, as compared with non-Hispanic white women living in other parts of California. Other breast cancer risk factors, including use of combined estrogen and progestin HT in 2003, physical inactivity, and obesity in women 50 years old or older, were less common in SFBA women or were similar in SFBA women and women in the rest of California and are therefore unlikely to have contributed to the higher incidence rates of breast cancer in the SFBA than in the rest of California.

The most notable risk factor changes we observed from 2001 to 2003 were the 76% and 72% relative decreases in the percentages of women reporting use of combined estrogen and progestin HT in the SFBA and the rest of California, respectively. Other risk factors did not appear to change substantially during this time period, but education level and mammographic screening history did increase modestly. Decreases in HT use are comparable to those noted in our recent report of a 68% decrease in the use of HT among middle-aged Northern California women after 2002 [10] and are consistent with findings in the US [12,38] and Germany [11]. Increasing HT use from 1994 to approximately 1999, the plateau in use from 1999 to 2001 following the 1998 release of findings from the Heart and Estrogen/Progestin Replacement Study [39,40], and the dramatic decrease in Northern California [10] after the 2002 WHI findings [14] closely mirror the trends we observed in both invasive breast cancer and breast cancer *in situ *incidence in the SFBA and the rest of California. This pattern, in addition to the decreases in ER^+ ^tumors, though limited by the high percentage of missing values, further supports the notion of a strong influence of the population prevalence of HT use on breast cancer incidence patterns.

It is unclear to what extent mammographic screening patterns, which have been associated with breast cancer incidence increases in the US (particularly in the late 1980s and early 1990s [30,38,41,42]), explain the elevated incidence rates in the SFBA. Our observations of higher incidence rates of breast cancer *in situ*, detected exclusively by mammography, and excess rates of localized and regional disease or tumors less than 2 cm, as well as excesses in women targeted by mammography screening guidelines (40 years old or older), suggest that the SFBA excess could be due in part to higher levels of screening, a finding supported by CHIS data that find a somewhat higher prevalence of mammographic screening in 2001, but not 2003, in the SFBA. The continued assessment of future trends in incidence rates will help us to understand whether a plateau in mammography screening [13,38] is playing a role in the observed trends.

Some, but not all [7], prior studies using ecologic and cohort study designs have found that sociodemographic characteristics [5,7] and risk factor distributions [4] explained the higher incidence rates of breast cancer in the SFBA compared with other regions. However, without information on residential mobility, these studies could not address the reasons why high-risk populations concentrate in certain geographic areas. Demographic change in the US appears to be favoring the migration of educated workers into certain geographic areas, including the SFBA, out-migration of less-educated persons to other parts of the country, and migration of service workers to suburbs on the periphery of the educated urban cores [43]. These patterns of migration are supported by 2000 to 2004 census data that list San Francisco and San Jose among the slowest-growing metropolitan areas and that list metropolitan areas outside the Greater SFBA as some of the fastest-growing [44]. The extent to which these migration patterns concentrate women with multiple established breast cancer risk factors in particular areas over time may help explain past and future breast cancer incidence trends. The breast cancer incidence patterns observed in SFBA women may be representative of patterns occurring in subpopulations with high breast cancer incidence, but for whom routine surveillance is challenging, such as women residing in West Los Angeles [45], women of high socioeconomic status [46], or female teachers in California [47]. Cancer surveillance efforts are further limited by the lack of population counts defined by individual characteristics, such as educational attainment within small geographic areas, that are necessary for estimation of incidence rates stratified by these characteristics. Therefore, non-Hispanic white women in the SFBA, where surveillance is ongoing, may serve as bellwethers for cancer trends occurring in similar subpopulations living in more heterogeneous areas.

A limitation of this analysis is that the data are ecological. That is, the data are available only at a geographic (that is, county) level rather than at the individual level. In addition, we did not adjust breast cancer incidence rates for known risk factors, nor did we have population prevalence estimates of risk factor changes over the 17-year study period or in the rest of the SEER regions. We were able to present risk factor changes between variables measured similarly in the 2001 and 2003 CHISs, but these data are limited by low response rates that could result in selection bias. However, response rates in CHIS were comparable to those in other population-based surveys [29], and response rates among the population-based controls included in the present analysis, though not directly comparable to CHIS, were higher. Even with these limitations, the ability to examine the prevalence of established breast cancer risk factors from two population-based sources allowed us to compare the prevalence of breast cancer risk factors in SFBA non-Hispanic white women with similar women in the rest of California. Furthermore, our data provided sufficient power for examining incidence trends by age, histologic subtype, stage at diagnosis, and hormone receptor status.

## Conclusion

Understanding breast cancer incidence and risk factor prevalence patterns in SFBA non-Hispanic white women informs the study of breast cancer etiology in two ways. First, future population-based studies attentive to exposure heterogeneity might include SFBA counties together with other US or international populations with lower documented incidence rates of breast cancer, in which established or putative risk factors may be less common, and protective factors may be more common. Second, high-incidence populations are also useful for the study of potentially important, yet poorly studied, community-level or cultural influences on breast cancer occurrence. A Wisconsin study [48], for instance, found that breast cancer risk was associated with high community socioeconomic status after adjustment for individual-level education and other established risk factors, suggesting that living in affluent communities impacts breast cancer risk above and beyond the risk conferred by individual risk factors. Thus, documenting breast cancer occurrence patterns in high-incidence regions, such as the SFBA, continues to be an activity of importance to breast cancer prevention efforts.

## Abbreviations

APC = annual percent change; BMI = body mass index; CHIS = California Health Interview Survey; ER = estrogen receptor; HL = Hodgkin lymphoma; HT = hormone therapy; ICD-O-3 = International Classification of Diseases-Oncology, 3rd edition; NCI = National Cancer Institute; SEER = Surveillance, Epidemiology, and End Results; SFBA = San Francisco Bay area; WHI = Women's Health Initiative.

## Competing interests

CC has served as an expert witness for plaintiffs in hormone therapy tort litigation. The other authors declare that they have no competing interests.

## Authors' contributions

MW, EJ, SG, and PH-R contributed control data from the Marin County Breast Cancer Study of Adolescent Factors, the San Francisco Bay Area Breast Cancer Study, the Reproductive Factors in Hodgkin Lymphoma in Women study, and the Bay Area Thyroid Cancer Study, respectively, and participated in the interpretation of data and critical review of the manuscript. EC participated in the interpretation of data and drafting and critical review of the manuscript. TK and CC created the study design, conducted the acquisition, interpretation, and analysis of data, and led the writing and review of the manuscript. All authors read and approved the final manuscript.
